# Awareness, Knowledge and Risky Behaviors of Sexually Transmitted Diseases among Young People in Greece

**DOI:** 10.3390/ijerph181910022

**Published:** 2021-09-23

**Authors:** Chrysa Voyiatzaki, Maria S. Venetikou, Effie Papageorgiou, Fragiski Anthouli-Anagnostopoulou, Panagiotis Simitzis, Dimitrios I. Chaniotis, Maria Adamopoulou

**Affiliations:** 1 Laboratory of Molecular Microbiology & Immunology, Department of Biomedical Sciences, School of Health and Care Sciences, University of West Attica, 12243 Athens, Greece; panosimis@gmail.com (P.S.); maryadam@uniwa.gr (M.A.); 2 Laboratory of Anatomy-Pathological Anatomy & Physiology Nutrition, Department of Biomedical Sciences, School of Health and Care Sciences, University of West Attica, 12243 Athens, Greece; mvenet@uniwa.gr (M.S.V.); fanthouli@uniwa.gr (F.A.-A.); dchaniotis@uniwa.gr (D.I.C.); 3 Reliability and Quality Control in Laboratory Hematology, Department of Biomedical Sciences, School of Health and Care Sciences, University of West Attica, 12243 Athens, Greece; efipapag@uniwa.gr

**Keywords:** sexually transmitted diseases, awareness, knowledge score, Greek young adults

## Abstract

Sexually transmitted diseases (STDs) affect mainly young individuals and cause health, social, and economic problems worldwide. The present study used a web questionnaire to assess the awareness, knowledge, sexual behaviors, and common practices regarding STDs in young Greek adults. The 1833 individuals, aged 18–30 years, who responded to the study seem to be particularly knowledgeable regarding STDs such as AIDS (97.7%), warts (97%), Chlamydia (92.2%), genital herpes (89.9%), syphilis (81.9%), and gonorrhea (72.1%), whereas lower percentages were noted for trichomoniasis (39.3%), Molluscum contagiosum (12.9%), mycoplasmosis (11.6%), and amoebiasis (7.4%). Regarding oral STD transmission, participants replied correctly for genital herpes (45%), warts (35.8%), and AIDS (HIV; 33.8%), whereas 30.2% were unfamiliar with oral sexual transmission. Of the participants, 52% were not aware that STDs might cause infertility. Only 40.4% of the respondents reported always using condoms during sexual intercourse, and 48.6% had never been tested for STDs. The majority of the young population (55%) presented a moderate knowledge STD score (41–60%) and was associated with demographic parameters such as age, gender, sexual preference, number of sexual partners, and residence (*p* < 0.05). These findings provide important information regarding the prevention of STDs and highlight the significance of developing more effective sex education programs for young people in Greece.

## 1. Introduction

Sexually transmitted diseases (STDs) are a major global health problem, with more than 340 million new cases occurring every year worldwide [1]. These infections are caused by bacteria, viruses, or protozoa and are transmitted mainly through sexual activity, although other routes of transmission, such as blood products, are also possible. In 2016, 376 million new infections were reported that included the following four STDs: trichomoniasis (156 million), Chlamydia (127.2 million), gonorrhea (86.9 million), and syphilis (6.3 million) [2]. In addition, >500 million subjects have been reported to have a genital infection by herpes simplex virus (HSV), and >290 million women with human papillomavirus (HPV) infection are estimated annually [3]. STDs cause severe consequences in the health of the affected population and are responsible for the development of various secondary diseases and complications, such as cervical cancer, infertility, pregnancy-associated complications including fetal mortality, increased risk for acquiring human immunodeficiency virus (HIV), and reduced quality of life due to psychological and social factors [4,5,6].

Although STDs are present in individuals of various age groups, they most frequently affect young adults since they represent non-homogenous populations with regard to their social needs and sexual behavior and are associated with various biological and social factors around transitioning from adolescence to adulthood [7]. Specifically, the premature initiation of sexual activity, the number of sexual partners, the need for self-assurance, the access to healthcare services, the financial status, and the education and occupation profiles are all factors associated with the increased frequency of STD infection in young individuals [8].

Teenagers are more prone to have relationships with multiple sexual partners, which in turn increases the possibility of STD infection [9]. It has been suggested that young people do not possess sufficient knowledge regarding STD infections and that their education and knowledge level are the main contributing factors affecting the incidence of infection [10,11]. A previous study in the United States indicated that 71students exhibited poor knowledge of the risk of developing STDs, while 50% of them were concerned about the risk of infection during sexual intercourse [12]. Moreover, it has been shown that although condoms are the most usual prevention measure for reducing the STD infection rate, their use among young adults is mainly focused on preventing unwanted pregnancy [13].

The reduction of STD infection in young adults remains a major challenge for health professionals in various countries. Ensuring that this target group is well informed of the risks and prevention measures against infection is imperative in order to reduce the dissemination of these diseases across the population. To date, a limited number of studies have explored the association of knowledge score and sexually transmitted diseases in Greece. The majority of the studies have explored the association of one specific sexually transmitted disease, such as HPV [14], HIV [15,16], or warts [17], with awareness, knowledge, behavior, and epidemiological characteristics in specific target groups such as men who have sex with men or health professionals. The current study advances further to examine a plethora of STDs in a population that was stratified into several subgroups based on age, sexual preference, residence, employment status, education, and gender. The overall aim of the present study was to assess the knowledge level of a Greek population of young adults regarding the risk of STD infection, the ways of transmission, and its diagnosis and prevention. A questionnaire was prepared, and the included subjects provided their answers online. This level of knowledge was compared with certain socio-demographic characteristics in order to identify potential trends that may lead to an increased STD infection rate.

## 2. Materials and Methods

### 2.1. Study Design and Participants

The present study was an observational cross-sectional study that aimed to determine the knowledge and educational levels of specific target groups regarding STDs. Moreover, information regarding the sexual behavior-associated risk was gathered from the participants. The current study included an online questionnaire that comprised 35 questions on demographic information, knowledge, trends, and common practice regarding STDs. The following inclusion criteria were used: (i) written consent for participation in the study, data analysis, and publication of the results; (ii) participants must be 18 years or older, and (iii) must be sexually active. The questionnaire was divided into the following main sections: (i) general demographic characteristics (10 questions); (ii) participant interest in STDs and prevention measures (4 questions); (iii) STD-associated knowledge and prevention measures (9 questions), and (iv) evaluation of sexual behavior-associated risk (12 questions).

#### Ethics Approval

The questionnaire was approved by the Ethics committee of the University of West Attica, and the relevant approval number was obtained (60515/08-09-2020). The relevant informed consent forms were provided to all participants to inform them of the aim of the present study.

### 2.2. Data Collection and Processing

This survey was conducted from 20 September 2020 to 31 December 2020. The questionnaire was designed by common Microsoft Office forms that fully complied with the General Data Protection Regulation so as to ensure the anonymity of the participants and exclude the possibility of their identification. The questionnaire was forwarded via social media platforms and via the academic community of the University of West Attica in Greece in order to facilitate its completion from a random population sample. A contact email (stdstudy@yahoo.com) was provided in the introductory note prior to completing the questionnaire, and a thank you note was provided following its completion. Following data collection, a database was established in Excel containing the information provided by the participants. The data were presented using descriptive statistics as absolute numbers (*n*) and percentages (%).

### 2.3. Evaluation of Knowledge Score

The knowledge score was calculated by the number of correct answers provided for each one of the seven following sections: (i) mode of STD transmission, (ii) diseases classified as STDs, (iii) STDs causing genital cancer, (iv) STDs causing infertility, (v) STDs transmitted by oral contact, (vi) protection against STDs with the use of condoms, (vii) protection against STDs with the use of contraceptive pills. Each correct answer was scored with one point and each incorrect answer with a zero. The total score was estimated by the sum of all points, corresponding to the number of correct answers for all seven questions. In case the number of answers was lower and/or equal to the maximum number of right answers, the knowledge score was estimated by the following formula:

Knowledge score of STD = number of right answers/maximum number of right answers × 100.(1)

In case the number of answers was higher than the maximum number of right answers, the knowledge score was estimated by the following formula:

Knowledge score of STD = number of right answers/number of answers × 100(2)

### 2.4. Data Management

The maximum time of data storage is anticipated to be >3 years from the study initiation since the study results have not been published yet. Following this time period, the collected data will be deleted from storage files, such as hard drives, USBs, and rewritable DVD discs. Specific software, such as file erasers and file shredders, will be used to remove data from online file hosting services, such as OneDrive. Electronic data will be deleted by formatting the relevant storage medium. All backup copies regarding these data will also be deleted.

### 2.5. Statistical Analysis

The data were expressed as mean ± standard deviation (S.D.) for continuous variables and as frequencies (*n*) and percentages (%) for categorical variables. Unifactorial analysis was performed using the Student’s t-test, one-way ANOVA, and Pearson’s correlation to examine the association between the outcome variable (knowledge score of STD) and the quantitative, qualitative demographic, and clinical characteristics. All demographic and clinical variables in the unifactorial analyses were included in a multiple linear regression model, using the enter method to determine the most significant independent factors associated with the outcome variable. The assumptions of the linear regression analysis (homoscedasticity, linearity, normality, and independence of error terms, as well as multicollinearity of independent variables) were examined. All tests were two-sided. *p* < 0.05 was considered to indicate a statistically significant difference. All analyses were carried out using the statistical package SPSS V21.00 (IBM Corporation).

## 3. Results

### 3.1. Socio-Demographic Characteristics of Respondents

The present study included 1833 participants that responded to the questionnaires submitted online. The majority of the respondents were females (75.1%), and the average age of all subjects was approximately 22 years. It is also interesting to note that a very high percentage of the respondents, including both males and females, were university students (94.3%) and city residents (85.8%) as opposed to subjects with lower education levels (0.9 and 4.8%) and residents of smaller towns (8.6%) and villages (5.7%). Moreover, a relatively high percentage of subjects reported heterosexual preference in sex partners (83.5%), whereas the average age of first sexual contact was 17.41 ± 1.92. The exact demographic characteristics are presented in Table 1.

### 3.2. Sexual-Risk Associated Knowledge

Following the recording of the demographic data, the subjects were assessed with regard to their knowledge and educational level (Primary or High School and University level) on STD infections and prevention methods. High percentages of the subjects were noted regarding knowledge on common STDs, such as AIDS (HIV; 97.7%), warts (97%), Chlamydia (92.2%), genital herpes (89.9%), syphilis (81.9%), and gonorrhea (72.1%) (Table 2). High percentages were also reported for hepatitis B (57.1%) and C (59.4%), followed by somewhat lower percentages for hepatitis A (31.4%) and pubic lice (29.4%) (Table 2). The lowest percentages were noted for COVID-19 (3.1%) (Table 2). When participants were queried on their knowledge of diseases related to genital cancer, the majority replied positively for HPV (51%), AIDS (17.9%), and genital herpes (12.1%), whereas a high percentage (43.2%) did not have sufficient knowledge to address this question (Table 2). Almost half of the participants (52%) included in the study were not aware that STDs could cause infertility. Specifically, Chlamydia (22.8%), AIDS (21.1%), syphilis (17.3%), gonorrhea (17.6%), genital herpes (12.8%), and Mycoplasmosis (5.1%) were among the main STDs that were less known for causing infertility (Table 2). The percentage of subjects who responded correctly was considerably lower (30.2%) when they were asked whether STDs could be transmitted by oral contact. The percentages of the subjects who replied correctly that certain STDs, such as genital herpes (45%), warts (35.8%), and AIDS (HIV, 33.8%) could be transmitted orally, were a bit higher (Table 2). Lower percentages were noted for syphilis (19.8%), gonorrhea (15.9%), and Chlamydia (17%) (Table 2).

The young population examined was more knowledgeable regarding the way of STD transmission and high percentages provided correct answers for vaginal (97.2%), anal (86.1%), and oral (76.7%) contacts, as well as blood transmission (68.1%) (Table 3). Lower percentages were noted for skin contact (28.3%), common utensils (8.5%), and clothing (9.1%) (Table 3). Moreover, a relatively high percentage of subjects (29.7%) replied incorrectly regarding the use of condoms and the prevention of STD transmission, whereas 65.4% provided correct answers (Table 3). However, regarding the use of contraceptive pills for the prevention of STD transmission, 84.2% of the respondents provided correct answers (Table 3), and only 4.7% responded incorrectly (Table 3).

### 3.3. Evaluation of Sexual Behavior-Associated Risk

The evaluation of sexual behavior-associated risk was performed by a questionnaire including the three following questions: (i) Do you use condoms? (ii) How often do you test yourself for STDs, and (iii) Do you have a previous history of STDs? A considerably low percentage of the participants reported using condoms during sexual intercourse (40.4%), whereas the majority reported using condoms in permanent sexual relationships (27.9%) and/or following testing of their partner for STD diagnosis (27.2%) (Table 4). The participants were queried on the frequency of conducting tests for STD diagnosis, and the majority of the respondents (48.6%) reported that they had never been tested for these types of diseases, whereas 23% reported testing at regular intervals following blood examinations (Table 4). Lower percentages were noted for other subcategories, such as at regular intervals following physical and blood examinations (10.6%), following suspicious sexual activity (5.7%), and following suspicion of STDs due to associated symptoms (12.1%). Finally, the majority of the participants reported that they have never had a previous history of STDs in their life, whereas 10% reported having a previous history of STDs (Table 4).

### 3.4. Assessment of STD Knowledge Score

**Figure 1:**
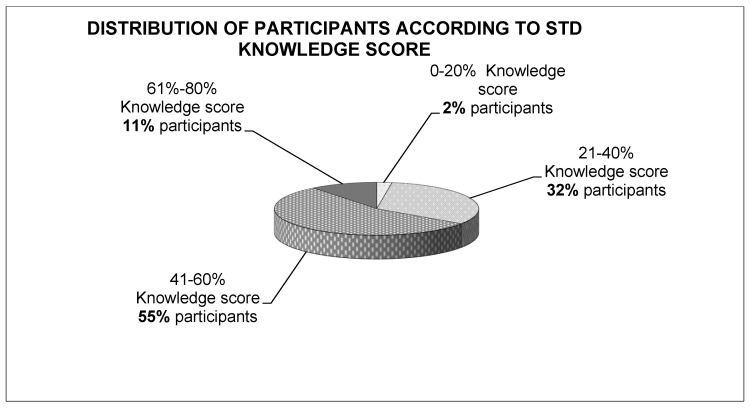
Distribution of participants according to STD knowledge score.

The knowledge STD score of the participants was categorized as low (0–40% knowledge score), moderate (41–60% knowledge score), and high (61–100% knowledge score). The majority of the participants (55%) presented a moderate knowledge score, followed by individuals with a low knowledge score (34%) and high knowledge score (11%) (Figure 1). The highest score was 80%.

### 3.5. Unifactorial Associations of Knowledge Score with Socio-Demographic Characteristics

The results of the knowledge score analysis were compared with certain socio-demographic parameters using unifactorial analysis. Significant differences were noted for gender, employment, residence, age, permanent partner relationships, and number of sexual partners (Table 5). With the exception of residence, the remaining parameters exhibited highly significant differences (*p* < 0.001) with the mean knowledge scores estimated (Table 5). Specifically, the unifactorial analysis revealed that females (*p* < 0.001), subjects with full-time jobs (*p* < 0.001), city residents (*p* = 0.039), and those subjects with permanent partner relationships (*p* < 0.001) presented higher STD-associated knowledge scores compared with that of males, those with part-time jobs or those who were unemployed, those who lived in towns or villages and those without a permanent partner, respectively. The parameters age (*p* < 0.001; *r* = 0.177) and number of sexual partners (*p* < 0.001; *r* = 0.105) correlated positively with the STD knowledge score, as determined by Pearson’s correlation analysis (Table 5).

### 3.6. Multifactorial Associations of Knowledge Score with Socio-Demographic Characteristics

A multiple regression model with enter method was employed to examine the independent contribution of the demographic variables to the STD knowledge score. Regression analysis accounted for 7.1% of the variance of the dependent variables [R^2^ = 0.071; F(8,1819) = 17.33, *p* < 0.001] (Table 6). According to the results, higher age (Beta ± SE: 0.62 ± 0.11; *p* < 0.001; R^2^ = 0.031), female gender (Beta ± SE: 4.62 ± 0.66; *p* < 0.001; R^2^ = 0.027), higher number of sexual partners (Beta ± SE: 0.08 ± 0.03; *p* = 0.003; R^2^ = 0.006), homosexual or bisexual preference (Beta ± SE: 1.90 ± 0.77; *p* = 0.014; R^2^ = 0.003), and permanent partner relationships (Beta ± SE: 1.23 ± 0.58; *p* = 0.035; R^2^ = 0.002) were significantly associated with higher STD knowledge score, while education (Beta ± SE: 2.39 ± 1.22, *p* = 0.060; R^2^ = 0.002), employment (Beta± SE: 0.80 ± 0.87, *p* = 0.420; R^2^ = 000) and residence (Beta ± SE: 1.01 ± 0.81, *p* = 0.215; R^2^ = 0.001) did not influence significantly the dependent variable. The results indicated that the increase in age for 1 year resulted in an increase in the knowledge score for 0.62, whereas female participants exhibited 4.62-fold higher knowledge scores than male participants (Table 6). Moreover, homosexual participants exhibited a 1.9-fold higher score than bisexual and subjects with a permanent partner had a 1.23-fold higher knowledge score than those without a permanent partner. Finally, an additional sexual partner (*n* + 1, where, *n* = number of sexual partners) caused an increase in the knowledge score of 0.08 (Table 6).

## 4. Discussion

STDs are responsible for causing various health, social, and economic problems worldwide. The majority of the affected subjects are young individuals, and this trend seems to be associated with their reduced knowledge and their lack of appropriate education on STD infection and prevention [10,11]. The present study was conducted for the first time in Greece and focused on addressing the lack of appropriate knowledge corresponding to young participants on a variety of STD infections and prevention. Specifically, we investigated the knowledge score and STD-associated education of 1833 Greek young adults who were interviewed online. The study group was stratified into several subgroups based on specific parameters, such as age, residence, education, employment, and sexual preference, so as to identify potential indicators that may be related to their educational level and knowledge score on STDs.

The analysis of the responses indicated that the highest percentages of the subjects were familiar with HIV, warts, Chlamydia, genital herpes, syphilis, and gonorrhea, followed by hepatitis B and C. HIV remains one of the most widely known STDs worldwide, as reported by previous studies performed in university students in Malaysia [18] and Turkey [19]. Hepatitis, gonorrhea, and syphilis have also been shown to be well-known STDs by previous studies [18,20,21,22]. A considerably high percentage of respondents were aware of Chlamydia (92.2%) causing STDs as opposed to a study involving 447 students in Brazil, which exhibited a lower percentage (15.8%) [22]. Similar findings to Brasil have been reported for the awareness and knowledge of adolescents regarding HIV/AIDS and HPV, and to some extent for Chlamydia in northern European countries, such as Russia, UK, Ireland, Finland, and Sweden [23]. The population examined in the current study responded positively to the majority of STDs queried. These findings may be attributed to the improved education of Greek students as opposed to other countries, as well as the contribution of social-educational campaigns that aim to provide additional information on the transmission, risks, and prevention measures of STDs. It must be stressed that sexual education programs in Greek schools are not available at the present time. Secondary school students are informed on the subject as part of the Biology teaching material. Social education campaigns are sometimes offered that aim to educate specific population groups. Although COVID-19 is not classified as an STD, only 5.5% of the participants reported that it is transmitted by oral contact, which is the main route of transmission mainly due to close contact or exposure to semen fluid. The fact that Molluscum contagiosum, amoebiasis, mycoplasmosis, and trichomoniasis exhibited considerably low percentages was possibly attributed to the limited information of the participants, and generally of the Greek population, regarding these diseases and their way of transmission, as opposed to the well-known STDs, such as HIV, hepatitis, Chlamydia, HPV, and genital herpes. Moreover, for some of these diseases, such as trichomoniasis, no screening guidelines have been previously reported, and the testing depends upon clinician practice, which is usually limited to symptomatic women [24]. However, for others, such as amoebiasis, which is mainly found among men who have sex with men (MSM), public health laboratory or pathologically diagnostic evidence is required for confirmed cases, and surveillance data may underestimate the exact disease burden [25].

Although trichomoniasis is a highly prevalent worldwide infection, it attracts little attention because most infected people do not show any symptoms, and no recommendations are available for general screening, so the surveillance of trichomoniasis has largely derived from general population-based and clinical-based studies [26].

Additionally, E. histolytica infection is associated with prevalent and incident HIV diagnoses suggesting new targets of surveillance and public health interventions for this sexually transmissible protozoan infection. Overall, the survey results showed that 39.3% and 7.4% percent of all questionnaire participants replied positively regarding knowledge on trichomoniasis and amoebiasis, respectively, indicating the urgent need to inform young people about the aforementioned STDs parasitic diseases [25].

Despite these encouraging findings, it is important to note that almost half of the young adults (52%) included in the study were not aware that STDs could cause infertility or could be related to genital cancers (43.2%). A previous study has demonstrated higher awareness of young individuals on the potential of Chlamydia to affect the male and/or female reproductive organs and lead to infertility compared with that noted for gonorrhea [27]. The young people who took part in the study of Goundry et al. in the UK were generally aware that STIs could lead to complications with fertility; however, knowledge about the process of how STIs cause infertility was poor [27]. The low awareness level of the Greek participants may be attributed to a lack of national sexual education programs, which are particularly active in other countries. For example, the National Chlamydia Screening Programme, set up in England and Wales, UK, in 2003, was a control and prevention program for sexually active individuals under the age of 25 [27].

With regard to the ways of STD transmission, vaginal, anal, and blood transmission were among the most relatively known ways of STD transmission in the Greek study population. This finding has been supported by previous studies [13,20], and high percentages of participants (>70%) were knowledgeable of the aforementioned routes of STD transmission. Moreover, although the majority (92%) of the participants knew that HPV viruses are sexually transmitted, only 35.8% of them were aware that the virus could be transmitted with oral contact as well, while 30.2% of the sample had no knowledge of oral transmission of the STDs. Other routes of transmission, such as clothing and skin contact, were associated with low percentages of participants responding correctly, possibly due to low dissemination of this information through the media (TV, internet). The majority of the participants responded correctly that the use of condoms and contraceptive pills does not protect against STDs. However, 29.7% of the respondents did not know that condom use does not totally prevent STD infection. This lack of knowledge has also been reported in a cross-sectional study comprising 542 undergraduate students, randomly selected from three Nigerian Universities [28]. The most common reasons given for the use of condoms were prevention of pregnancy (91.7%) and the ability to provide protection from STDs (89.1%) [28]. This suggested that these young adults were not aware that condom use does not prevent STDs [27]. However, a study involving 367 participants conducted in Brazil reported that the frequency of condom use with a stable partner was significantly proportional to condom use with a casual partner and to the access to information on prevention given that lack of access to prevention supplies and information accounted for respondent programmatic vulnerability [29]. In a study that examined the knowledge and awareness of adolescents in northern European countries (Sweden, Ireland, Russia, England, Ukraine, Croatia, Finland, and Germany), the participants reported that the use of condoms helps protect against contracting an STD, although some of them still regarded condoms primarily as an interim method of contraception before using the pill [23]. Therefore, other factors, such as the nature and number of sexual partners and the general education of the participants, may be responsible for this lack of knowledge.

With regard to STD prevention measures, it is important to highlight that approximately half of the participants had never been tested for STDs before, probably because of limited awareness and/or high cost, whereas only 40.4% always used condoms during sexual intercourse. In a similar study performed in 436 high school and university students in Italy, low rates of correct responses were achieved when the participants were asked about the frequency of HIV testing and their risk of exposure, possibly due to the lack of an appropriate health education program [30]. In addition, several factors have been previously proposed for the lack of condom use during sexual intercourse, such as long-term relationships, the need to please the partner, lack of knowledge of the benefits, the perception that serious STDs can now be controlled with medication, the influence of tradition, and the refusal of the partner [31].

A similar percentage was reported for 1017 first- and fourth-year students from the University of Pristina in Kosovo regarding the use of condoms following their most recent sexual encounter and encounters with casual partners [32]. The factors associated with condom use following recent sexual encounters were gender, the use of medical sources of information about HIV, having a positive approach to HIV testing, and not having had a sexually transmitted infection [32]. These data corroborate some of the findings of the present study. Poor use of condoms and a high number of partners have also been reported as the main factors responsible for the incidence of sexually transmitted infections in an Italian group comprising 294 subjects with STIs [33], suggesting that condom use is not always adopted by young individuals during their sexual activities.

The knowledge score of the participants was estimated based on the number of correct answers divided by the total number of correct answers. The data indicated that more than half of the participants (55%) exhibited an average knowledge score range (41–60%). The mean knowledge score was estimated to be 44.65 ± 12.5. This suggested that this percentage replied correctly to almost half of the questions, which implied a substantial lack of knowledge regarding sexual behavior-associated risk, prevention, and infections. This knowledge score was associated with specific parameters such as gender, employment, residence, age, and permanent partner relationships. The fact that females are more knowledgeable than males regarding STDs has been previously demonstrated [18,34] and the findings presented in the current study corroborate this conclusion. In addition, age is the main factor that contributes to an increased knowledge score since young individuals are less mature and more prone to adopt risky and/or careless sexual behavior. In this study, the age group 25–30 presented a statistically significant higher knowledge score compared to the 18–24 age group. Young people (10 to 24 years of age) experience a transition period in their life during which sexual behaviors and decisions are being shaped [20,35]. Moreover, many young individuals frequently enter an active sexual life with unprotected intercourse without considering the consequences, whereas others experience sexual intercourse forcibly [20]. Under these circumstances, their decisions are not the results of critical thinking and/or mature behavior, and these young adults do not necessarily take into consideration their lack of appropriate knowledge and education on sexual behavior-associated risks and prevention measures. Therefore, it is considered that as age increases, the knowledge, awareness, and education of these individuals on STDs will also increase.

Furthermore, residence and employment status were not associated with an increased knowledge score, as determined by the multivariate analysis, whereas when these parameters were independently compared with the outcome using univariate analysis, highly significant associations were found. Previous studies have shown that residence, in combination with other factors such as gender and younger age, is associated with a higher incidence of STDs [36,37]. However, in the current study, residence and employment status were not associated with the knowledge score of the participants when all the parameters were assessed together. This may be explained by residence and employment status being affected by gender, education, sexual preference, and the incidence of a single partner relationship, which may affect the knowledge score and mask potential significant associations with residence and employment status that were noted when the latter variables were assessed independently.

Finally, highly significant associations were noted with regard to the knowledge score and sexual preference, the number of sexual partners to date, and the relationship with a permanent partner. It was found that homosexual subjects were almost two times more knowledgeable than heterosexuals. A similar finding has been reported previously in a group of US medical students [38]. The parameter sexual orientation was associated, among other parameters, with high knowledge score of the subjects investigated, and students identified as non-heterosexual (i.e., homosexual, bisexual, asexual, or pansexual) scored significantly higher than heterosexual students [38]. This may be due to homosexuals having considerably more lifetime sexual partners in comparison to heterosexuals, which in turn may result in increased sex-related knowledge, as reported previously [39]. Moreover, the number of sexual partners may indeed increase the knowledge score by sharing information among different individuals and by acting as an informal educational process for friends and partners via their internal communication. It has been proposed that friends may facilitate everyday conversations about health and STIs by sharing stories and advice and discussing the challenges of navigating relationships in young individuals living in Australia for ≥3 years [40]. Moreover, an early study has suggested that the trust towards a sexual partner may influence his/her STI status and corresponding knowledge state [41]. A permanent relationship may lead to a more stable sexual life that can increase prevention measures and the knowledge score on STDs.

The present study contains no limitations regarding ethnic origin, religion, migration status, or other specific population subgroups in Greece.

The questionnaire was posted on Google platform and social media, and it was available to individuals 18 years or older who can understand/speak/write the Greek language.

## 5. Conclusions

The present study provided a comprehensive analysis of the knowledge score and educational level of a Greek population comprising young individuals regarding STDs and their associated risks and prevention measures. The data indicated that the majority of the studied population was aware of the main STDs, including HIV, Chlamydia, syphilis, gonorrhea, genital herpes, and hepatitis, as well as of the main routes of STD transmission. A considerably high percentage of the subjects were aware that condom and contraceptive pill use do not protect against STD infection, although approximately 30% of the subjects responded incorrectly that condom use protected against STD infection. The knowledge score of the participants was considered average (45.97 ± 13.92) as only a small percentage responded correctly to the majority of the questions (0.9% replied correctly for 81–100% of the questions) asked. Significant factors associated with the knowledge score were determined and included gender, age, residence, employment, number of sexual partners, and permanent partner relationship. In order to improve the knowledge score and STD awareness of these subjects, the relevant authorities need to develop a systematic plan of sexual education at school. It has been reported that health education programming for youth in Greek schools is imperative to promote healthier lifestyles and to prevent chronic and infectious diseases [42]. Moreover, national programs can be implemented to increase the education of young individuals on STDs. It is important to note that recently the ministry of education has declared compulsory training on sexual education at schools in Greece. Effective dissemination of relevant scientific knowledge is also required as well as modification of incorrect beliefs and concepts regarding STDs that are present in the vast majority of the young population. Finally, the financial status of Greece is a contributing factor that cannot be dismissed in assessing the level of STD awareness. It is important to focus on promoting the sexual education of adolescents and young adults. Counseling, education, and information are significant prevention measures to be used against the spread of sexually transmitted diseases. Moreover, relevant research studies must focus on the evaluation of the effectiveness of sex education and its social impact on the affected population. By combining the aforementioned measures, a more complete and substantial level of sex education can be accomplished, leading to the prevention of relevant health and social problems.

## Figures and Tables

**Table 1:** Demographic characteristics of the population examined.

Age: Mean ± SD (Min–Max)	21.48 ± 2.88(18–30)
**Gender**: male/female/other *n* (%)	449(24.5)/1377(75.10/7(0.4)
**Education** Primary/High School/University, *n* (%)	16(0.9)/88(4.8)/1729(94.3)
**Employment**: Full-time/Part-time/Unemployed, *n* (%)	281(15.3)/417(22.7)/1135(61.9)
**Residence**: City/Town/Village, *n* (%)	1572(85.8)/157(8.6)/104(5.7)
**Do you have a permanent partner**: no/yes, *n* (%)	848(46.3)/985(53.7)
**Sexual preference**: Heterosexual/Homosexual/Bisexual, *n* (%)	1530(83.5)/86(4.7)/217(11.8)
**Number of sexual partners to date**: Mean ± SD (min–max)	5.37 ± 10.32(0–250)
**Age of 1st sexual contact(*n* = 1322)**: Mean ± SD (min–max)	17.41 ± 1.92(10–29)

**Table 2:** Knowledge of the young population regarding STDs and prevention methods.

	Diseases Classified as STDs	Diseases Causing Genital Cancer	Diseases Causing Infertility	Diseases Transmitted by Oral Contact
WartsHuman papilloma virus (HPV)	**1778 (97.0)**	**935 (51.0)**	467 (25.5)	**657 (35.8)**
Trichomoniasis	**720 (39.3)**	31 (1.7)	87 (4.8)	**66 (3.6)**
Hepatitis C	**1089 (59.4)**	147 (8.0)	169 (9.2)	264 (14.4)
Hepatitis B	**1047 (57.1)**	124 (6.8)	161 (8.8)	265 (14.5)
Hepatitis A	575 (31.4)	54 (2.9)	75 (4.1)	**135 (7.4)**
Genital Herpes	**1647 (89.9)**	**222 (12.1)**	**234 (12.8)**	**825 (45.0)**
Infectious mononucleosis	292 (15.9)	20 (1.1)	57 (3.1)	235 (12.8)
COVID-19	57 (3.1)	5 (0.3)	7 (0.4)	100 (5.5)
Malaria	20 (1.1)	2 (0.1)	12 (0.7)	18 (1.0)
AIDS (HIV)	**1790 (97.7)**	**329 (17.9)**	**386 (21.1)**	620 (33.8)
Mycoplasmosis	**212 (11.6)**	24 (1.3)	**94 (5.1)**	58 (3.2)
Syphilis	**1502 (81.9)**	145 (7.9)	317 (17.3)	**362 (19.8)**
Gonorrhea	**1321 (72.1)**	93 (5.1)	**323 (17.6)**	**292 (15.9)**
Amoebiasis	**136 (7.4)**	14 (0.8)	35 (1.9)	44 (2.4)
Echinococcosis	135 (7.4)	15 (0.8)	40 (2.2)	32 (1.8)
Molluscum contagiosum	**236 (12.9)**	16 (0.9)	31 (1.7)	83 (4.5)
Chlamydia	**1690 (92.2)**	182 (10.0)	**418 (22.8)**	**312 (17.0)**
Pubic lice	**539 (29.4)**	25 (1.4)	61 (3.3)	38 (2.1)
Do not know		792 (43.2)	953 (52.0)	553 (30.2)

All variables are presented as numbers (percentages) of answers. **Bold font** indicates the correct answer.

**Table 3:** Knowledge of the young population regarding STDs and prevention methods.

Way of Transmission		The Use of Condoms Protects Against STDs		The Use of Contraceptive Pills Protects Against STDs	
**Vaginal contact**	**1782 (97.2)**	**No**	1198 (65.4)	Correct	86 (4.7)
**Anal contact**	1578 (86.1)	Yes	545 (29.7)	**No**	1544 (84.2)
**Oral contact**	1406 (76.7)	Do not know	90 (5.0)	Do not know	203 (11.1)
**Blood**	1249 (68.1)				
**Clothing**	166 (9.1)				
Common utensils	156 (8.5)				
**Skin contact**	518 (28.3)				
Do not know	19 (1.0)				

All variables are presented as numbers (percentages) of answers. **Bold font** indicates the correct answer.

**Table 4:** Evaluation of sexual behavior-associated risk.

**In which cases do you not use a condom?**	
Never	14 (0.8)
Only if partner prefers it	23 (1.3)
In permanent sexual relationships	512 (27.9)
In permanent sexual relationships following testing of a partner for STDs	499 (27.2)
In case of HIV non-detectable seropositive partner	39 (2.1)
In case my partner or I am receiving PrEP treatment	5 (0.3)
Always	741 (40.4)
**How often do you test yourself for STDs?**	
At regular intervals following physical and blood examinations	194 (10.6)
At regular intervals following blood examination	422 (23.0)
Following suspicious sexual activity	105 (5.7)
Following suspicion of STDs due to associated symptoms	221 (12.1)
Never	891 (48.6)
**Do you have a previous history of STDs?**	
Yes (I always notify my partners):	112 (6.1)
Yes (I notify only my permanent partner but not my occasional partners):	56 (3.1)
Yes, without notifying my partners:	16 (1.0)
No	1.649 (90%)

**Table 5:** Unifactorial analysis of STD knowledge score in relation to demographic characteristics of the young Greek population.

		STD Knowledge Score	
		Mean ± SD (%)	*p* Value
Gender	Male	41.09 ± 12.16	<0.001
	Female	45.84 ± 12.36	
Education	Primary or High School	42.33 ± 12.08	0.051
	University	44.80 ± 12.52	
Employment	Unemployed	44.37 ^a^ ±12.52	<0.001
	Part-time	43.50 ^a^ ±11.99	
	Full-time	47.51 ± 12.81	
Residence	Town or Village	43.13 ± 12.92	0.039
	City	44.91 ± 12.42	
Do you have a permanent partner	No	43.44 ± 12.85	<0.001
	Yes	45.70 ± 12.12	
Sexual preference	Heterosexual	44.32 ± 12.59	0.065
	Homosexual	46.64 ± 12.66	
	Bisexual	46.21 ± 11.71	
Age	Pearson’s r	*r* = 0.177	<0.001
Number of sexual partners to date		*r* = 0.105	<0.001

^a^: *p* < 0.001 vs. full-time. SD: Standard Deviation

**Table 6:** Multifactorial analysis of STD knowledge score in relation to demographic characteristics.

	Reference Category	R^2^	Beta ± SE	*p*-Value
Age	---	0.031	0.62 ± 0.11	<0.001
Gender (female)	Male	0.027	4.62 ± 0.66	<0.001
Education (university)	Primary or High School	0.002	2.39 ± 1.22	0.050
Employment (Unemployed, -Part time)	Full time	0.000	0.80 ± 0.87	0.420
Residence (town-village)	City	0.001	1.01 ± 0.81	0.215
Do you have a permanent partner	No	0.002	1.23 ± 0.58	0.035
Sexual preference(Homosexual-Bisexual)	Heterosexual	0.003	1.90 ± 0.77	0.014
Number of sexual partners to date	---	0.006	0.08 ± 0.03	0.003

Beta: Beta coefficient of linear regression, SE: Standard Error of Beta coefficient.

## Data Availability

Data available on request due to restrictions eg privacy or ethical. The data presented in this study are available on request from the corresponding author. The data are not publicly available due to protection of privacy of the participants by all means.
